# Collagen Type I Conduits for the Regeneration of Nerve Defects

**DOI:** 10.3390/ma9040219

**Published:** 2016-03-23

**Authors:** Silvan Klein, Jody Vykoukal, Oliver Felthaus, Thomas Dienstknecht, Lukas Prantl

**Affiliations:** 1 Center for Plastic-, Hand- and Reconstructive Surgery, University Hospital Regensburg, Franz-Josef-Strauss-Allee 11, Regensburg 93053, Germany; oliver.felthaus@ukr.de (O.F.); lukas.prantl@ukr.de (L.P.); 2 Translational Molecular Pathology, University of Texas MD, Unit 951, 7435 Fannin Street, Houston, TX 77054, USA; jvykouka@mdanderson.org; 3 Department of Orthopaedic Trauma Surgery, University Medical Center Aachen, Pauwelsstrasse 30, Aachen 52074, Germany; tdienstknecht@ukaachen.de

**Keywords:** Type I collagen nerve conduit, biocompatibility, peripheral nerve repair

## Abstract

To date, reliable data to support the general use of biodegradable materials for bridging nerve defects are still scarce. We present the outcome of nerve regeneration following type I collagen conduit nerve repair in patients with large-diameter nerve gaps. Ten patients underwent nerve repair using a type I collagen nerve conduit. Patients were re-examined at a minimal follow-up of 14.0 months and a mean follow-up of 19.9 months. Regeneration of nerve tissue within the conduits was assessed by nerve conduction velocity (NCV), a static two-point discrimination (S2PD) test, and as disability of arm shoulder and hand (DASH) outcome measure scoring. Quality of life measures including patients’ perceived satisfaction and residual pain were evaluated using a visual analog scale (VAS). No implant-related complications were observed. Seven out of 10 patients reported being free of pain, and the mean VAS was 1.1. The mean DASH score was 17.0. The S2PD was below 6 mm in 40%, between 6 and 10 mm in another 40% and above 10 mm in 20% of the patients. Eight out of 10 patients were satisfied with the procedure and would undergo surgery again. Early treatment correlated with lower DASH score levels. The use of type I collagen in large-diameter gaps in young patients and early treatment presented superior functional outcomes.

## 1. Introduction

Although advances in microsurgery procedures have brought great progress for the treatment of nerve injuries, the limiting factor for the overall recovery of function remains the unsatisfactory results following nerve restoration [1,2,3]. Whereas digital nerves are frequently affected by hand injuries, more proximal lacerations of large-diameter nerves in the forearm region are rare but still severe injuries in clinical practice [4].

Ideally, prompt neurorrhaphy performed under minimal tension is the most promising treatment for such lesions [5,6]. However, existing nerve gaps following adequate debridement or extensive injuries frequently impede tensionless end-to-end coaptation, and nerve repair under tension seems to be prognostically adverse [5,6]. Although there is a general consensus that proper treatment for this dilemma involves bridging the defect zone, the method to achieve nerve continuity is a matter of ongoing debate [7,8]. Autologous nerve grafts, acellular nerve transplants, and nerve guidance conduits are commonly discussed in the context of peripheral nerve gap repair [9,10,11,12].

Autologous nerve segments can easily be harvested from sensory nerves and provide a viable source of Schwann cells, which are known to be invaluable for nerve regeneration [13]. Although nerve autografts are still considered the gold standard for peripheral nerve repair, disadvantages such as donor site morbidity, potential neuroma formation and mismatch in nerve quality and diameter have yielded novel approaches for the purpose of bridging nerve discontinuities [14,15,16]. Tubular nerve guidance conduits offer a straightforward option for surgical nerve repair that surmount the limitations of autologous nerve transplants. Nerve conduits are either obtainable as synthetic nerve guidance tubes from various biocompatible polymers or can be improvised as autologous transplants from ligated vessel segments [11,17]. Among the synthetic materials applied in human trials are collagen type I/III, acellular nerves, poly(d,l-lactide-caprolactone), polyglycolic acid and silicone [18]. The tubular space within the conduit, together with neurotrophic and neurotropic factors secreted by the nerve stumps, seem to provide a microenvironment that facilitates axonal sprouting and the formation of a glial sheath for sufficient nerve regeneration [11,17]. Recent studies using collagen conduits for the reconstruction of digital nerves present encouraging results [19,20]. Despite numerous experimental investigations, however, scarce clinical data exist to support the widespread use of guidance conduits, especially in the context of the enduring discussion as to whether or not large-diameter nerve reconstruction could be properly carried out using this approach [8,21]. In this study type I collagen conduits were implanted to bridge traumatic nerve discontinuities of less than 1.2 cm in lacerated nerves of the distal forearm region. The outcome was assessed by static two-point discrimination (S2PD), nerve conduction velocity relative to the uninjured limb, disability of arm shoulder and hand (DASH) outcome measure scoring, and patients’ perceived satisfaction.

## 2. Results

### 2.1. Implant Biocompability

No wound healing problems or signs of allergic reactions occured in any of the patients treated with type I collagen guidance conduits. No signs of host reaction were seen in any of these patients. No perioperative complications were recorded, and no conduit removal was required. No painful neuromas, hypertrophic or tender scars were noted. Each of the 10 patients was dismissed to the outpatient clinic with neither readmission nor surgical revision being necessary. These results support the biocompatible behavior of the employed conduits in conjunction with peripheral nerves.

### 2.2. Disability of Arm Shoulder and Hand (DASH) and Visual Analogue Scale (VAS)

**Figure 1:**
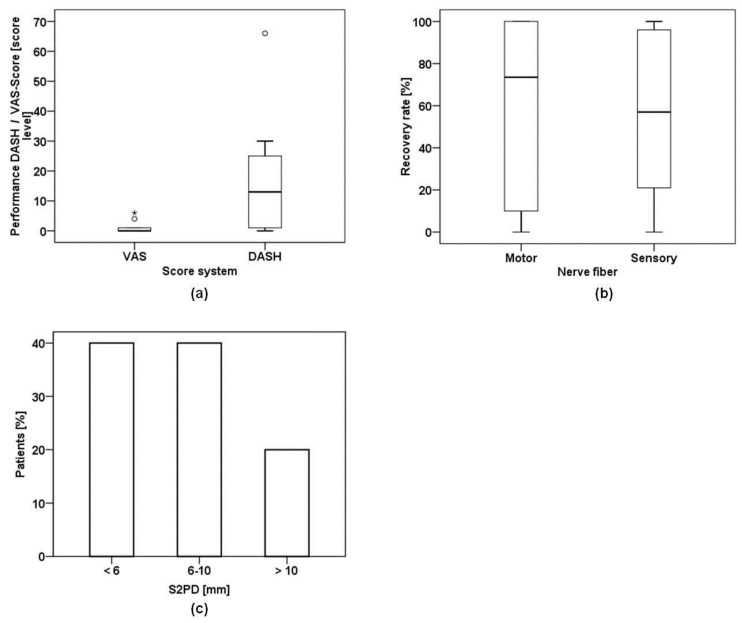
Outcome measurements after a mean follow up of 19.9 months post-surgery: (**a**) Median of DASH and VAS scores presented as box plots. The median DASH score was 13.0 with a mean of 17.0. The median in VAS scores was 0.0 with a mean of 1.1; (**b**) The box plot represents nerve function relative to the contralateral corresponding nerve. For motor function the median was 78.5%. For sensory function the median was 23.0%; (**c**) Bars represent percentage of patients with their corresponding results in the S2PD. Eighty percent of the patients reached less than 10 mm in the S2PD assessment.

The mean DASH score was 17.0 (range (R) 0–66, median (M) 13.0, standard deviation (SD) 20.6. The mean VAS score was 1.10 (R 0–6, M 0.0, SD 2.13) (Figure 1a). The mean DASH score level seems to be an acceptable outcome, considering the severity of such major motor nerve injuries [22].

### 2.3. Nerve Conduction and Static Two Point Discrimination (S2PD)

The nerve conduction velocity for motor responses presented a mean of 64.8% (R 0%–100%, M 78.5% and SD 39.95%) compared to the unharmed limb (Figure 1b). Forty percent of the patients reached less than 6 mm in the S2PD, 40.0% 6–10 mm and 20.0% more than 10 mm (Figure 1c). The clinical outcome in discriminative sensory evaluation yielded more promising results than suggested by the nerve conduction studies.

### 2.4. Patient-Related Confounding Factors

**Figure 2:**
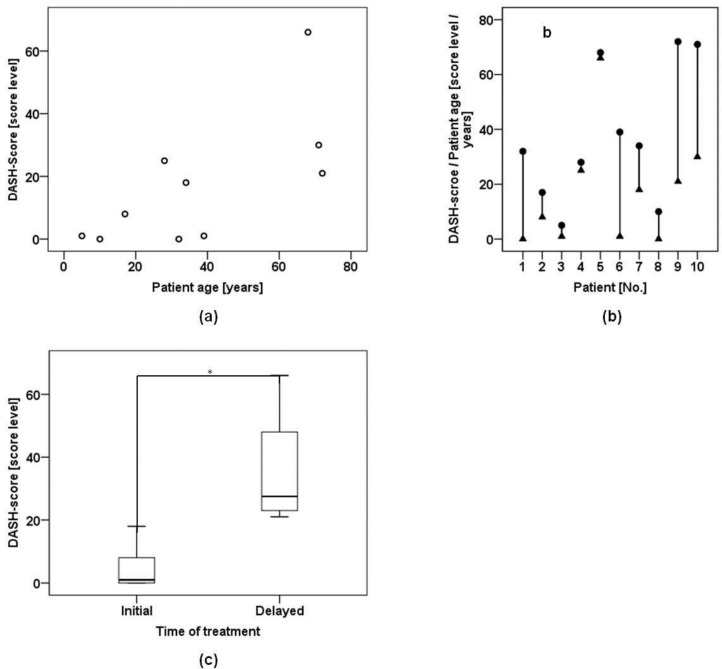
Confounding variables for the functional outcome after nerve injuries: (**a**) There was a positive correlation between patient age and DASH results, notwithstanding that this correlation was not statistically significant; (**b**) Plot shows DASH score level (triangles) and corresponding age (dots) of each patient. Younger patients tended to have lower DASH score levels; (**c**) Box plot represents DASH score results of patients who received either initial treatment or delayed treatment. Initial treatment resulted in better DASH score levels. This difference proved to be significant (*).

We found a positive correlation between patient age and DASH scores, however this correlation lacked statistical significance (*r*_s_ = 0.61, *p* = 0.06) (Figure 2a,b).

In contrast the difference in DASH-score levels between initially treated patients and patients who underwent delayed treatment was statistically significant (*p* = 0.01) (Figure 2c). Mann-Whitney testing indicated that DASH scores were higher for patients who underwent delayed treatment than in patients who underwent initial treatment (*U* = 0.0, *p* = 0.01). The mean DASH score in the initial treatment group was 2.0 (R 0–8, M 1.0, SD 3.4), whereas the delayed treatment group yielded a mean DASH score of 23.0 (R 18–66, M 25.0, SD 19.5). According to these results time of treatment seemed to determine the outcome more than the patient’s age at the time of nerve repair.

## 3. Discussion

Numerous recent studies have reported an acceptable clinical outcome regarding sensation with the use of collagen conduits in digital nerve repair [10,19,23,24,25], but few clinical studies exist in the literature reporting tubulization and achievable levels for motor nerve or mixed nerve injuries [8,21]. As large-diameter nerve injuries are rare in clinical practice, most authors tend to analyze their outcome after nerve repair along with data from small-diameter nerve injuries. Mackinnon *et al.* reported results ranging from good to excellent in the two-point discrimination of more than 80% of patients after sensory nerve repair in digits with defect lengths ranging from 0.5 to 3.0 cm using bioabsorbable polyglycolic acid tubes [25]. Most obviously, such promising results should not be expected in large-diameter nerves with mixed nerve fibers [21]. Still, pooling data from digital nerves with injuries of large-diameter nerves in the forearm region is likely to obscure the overall results after conduit repair in large diameter nerves [9,26].

In this study, we report on patients treated with type I collagen nerve conduits in the forearm region for defects in ulnar and radial nerves of up to 1.2 cm, with a special interest in functional recovery and patient satisfaction. Purified type I bovine collagen conduits were used with a wall thickness of under 0.5 mm, fabricated from bovine tendon material. This material is biodegradable within 36 months [27]. Among the available alternatives for synthetic biodegradable conduits are polylactic acid and caprolactone [28], whereas the use of non-biodegradable first-generation conduits (gelatin, agar, and silicon) could not be established in clinical practice [29]. Although polylactic acid seems to be functionally inferior compared to collagen and caprolactone, the optimal material and cellular compounds for biodegradable conduits are still subject of intensive research [28,30,31,32]. Furthermore, there has been frequent reporting on the use of conduits pre-seeded with Schwann cells, or pre-seeded with stem cells differentiated into Schwann cells [33,34]. However, none of these approaches have been implemented into clinical practice for various reasons. *Ex vivo* tissue processing, the application of cell transplants of heterologous origin and the lack of clinically approved conduit materials prohibit the routine use in patients, as does as the lack of long-term results. On the other hand, very few studies exist that used clinically approved materials in conjunction with autologous cells.

In this study the main outcome measure for motor function was the corresponding nerve conduction velocity and DASH score level. In our patient cohort, the mean recovery rate in motor nerve electrophysiology was 64.8%. The mean DASH score level of 17.0 seems to be an acceptable outcome, considering the severity of such major motor nerve injuries [22]. When we reported on the outcome of conduit repair in median nerve lesions, the overall DASH score levels were lower [20]. These difference in outcomes might be attributed to the older patient cohort and the inclusion of patients that underwent delayed nerve repair in this particular study [20,35]. Besides these incongruencies, the type of affected nerve has been identified as one prognostic factor for the rate of nerve recovery [35]. Unfortunately most studies using the DASH scoring instrument report solely on sensory nerve repairs, which impedes a straight comparison of this value [24,36]. The great variety of influencing factors associated with functional recovery in peripheral nerve repair impede the direct comparison of results with other reported trials [35].

Moore *et al.* reported on four cases of conduit failure in large-diameter nerves [21]. In this series different types of conduits were used, and the study included a variety of traumatic, iatrogenic injury patterns. Further, the lacerations were treated by different surgeons. Perhaps these variables confounded the clinical outcomes. In contrast Jardin *et al.* reported on another series of three cases that underwent conduit repair in discontinuities of mixed nerves in the forearm region, and found more encouraging results [37].

Marked loss in sensibility is one major cause for the poor outcome after large-diameter nerve injuries [38]. To monitor the outcome in hand sensibility, we measured nerve conduction of sensory nerve fibers. Additionally, S2PD was assessed to evaluate functional outcomes. We considered the mean recovery rate of 46.1% in sensory nerve conduction rather disappointing. Interestingly, the clinical outcome in discriminative sensory evaluation yielded more promising results. Here 80% of the patients showed S2PD of less than 10 mm (Figure 1d). This mismatch between functional recovery and neurophysiological studies has also been observed by other investigators [38], and might originate from the misdirection of regrowing axons in the regenerating nerve.

The time of nerve repair has been identified as a critical variable for the outcome after peripheral nerve repair [5,6,39]. Hence the patient cohort was divided into two subgroups for further analysis. One group received initial treatment, whereas the other group underwent delayed nerve repair. Here the outcome in DASH-score level was significantly better in the initial treatment group (Figure 2c). Further the regenerative potential is generally expected to be better in younger patients, as cell proliferation and tissue microperfusion is more effective than in elderly individuals [5,6]. When we analyzed DASH score level and patient age for their correlation, we did find a positive dependence in terms of higher DASH scores occurring in higher ages (Figure 2a). However this positive correlation failed to prove significance, when subjected to statistical analysis (Figure 2b).

## 4. Materials and Methods

### 4.1. Patient Cohort

Ten patients with nerve lesions of the radial and ulnar nerve in the forearm region underwent nerve repair using the tubulization technique with a type I collagen guidance conduit. Tensionless conventional nerve repair (e.g., end-to-end coaptation or neurorraphy) was impossible due to nerve discontinuities ranging from 0.8 to 1.2 cm in flexed joint position. The indication was acute nerve injury (injury < 7 days) in 5 cases and delayed nerve repair in another 5 cases. Nerve repair more than 7 days after the event of injury was considered as delayed repair and corresponding patients were allocated to the delayed treatment group for statistical analysis. The mean patient age was 37.6 years; 3 patients were of female sex and 7 patients were males. Mean follow up time was 19.9 months, with a minimal follow up of 14.0 months and maximum follow up of 24.0 months.

### 4.2. Nerve Conduit

A single type I collagen conduit (NeuraGen, Integra Life Sciences, Plainsboro, NJ, USA) was used to reconstruct the nerve defects. The dimensions of conduits used in this experiment were 4–7 mm in diameter and 2 cm in length. All conduits were used according to the manufacturer’s instructions.

### 4.3. Disability of the Arm, Shoulder and Hand Score 

The validated German translation of the DASH questionnaire was obtained from each patient as described previously [40]. DASH results are presented in a single score ranging from 0 (no disability) to 100 (maximum disability).

### 4.4. Nerve Conduction Velocities

For the sensory nerve conduction study, standard orthodromic sensory nerve action potentials were obtained by stimulating the digits involved and recording the responses evoked after supramaximal stimulation. Compound muscle action potentials were obtained using disk electrodes with the active electrode placed over the belly muscle and the indifferent electrode over the tendon. The same procedures were carried out on the uninjured opposite arm as a control.

### 4.5. Surgical Technique

All patients were operated on by experienced hand surgeons with microsurgical expertise. A perioperative single-dose antibiotic treatment (Cephazoline injection, 2 g) was administered intravenously before the tourniquet was activated. No relevant primary wound contamination occurred in any of the cases. Injured segments of the nerve were proximally and distally resected until no residual interfascicular scarring was seen. Surgeries were performed under tourniquet ischemia with a maximum tourniquet time of 120 min. in all patients; the tourniquet cuff was opened before insertion of the nerve fibers into the conduit to prevent blood pooling inside the conduit, which is known to inhibit nerve regeneration. The length of nerve damage was evaluated with all involved joints in a flexed position, and USP 9-0 polypropylene U-sutures were used to fasten the nerve ends to the conduit. For this step, the initial stitch was from outside through the tube and then through the epineurium and back through the tube wall in an in-to-outside direction. Two separate sutures were used at each end for proper positioning of the nerve in the tube. Saline solution was used to repeatedly rinse the conduit. For this procedure the nerve endings were inserted into the conduit by approximately 5 mm on each side. A plaster splint was applied after wound closure and left in place for 3 weeks.

### 4.6. Pain Assessment

Perceived levels of pain were evaluated by visual analog scale (VAS) measurements. VAS scores of 10 represented maximum pain, whereas scores of 0 denoted no pain on a 100 mm continuous scale.

### 4.7. Static Two-Point Discrimination

Results below 6 mm were graded as normal values in the static two-point discrimination (S2PD) assessment.

### 4.8. Statistical Analysis

Mann-Whitney-U-Test testing was performed for comparison of DASH scores in early *versus* late treatment groups. Spearman’s correlation was used for correlation analysis between patient age and outcomes represented by DASH scores. Analysis was performed using SPSS, Version 22 for Windows (SPSS, Inc., Chicago, IL, USA).

## 5. Conclusions

In conclusion, we report the outcome for the use of nerve conduits in patients with large-diameter nerve lacerations, with the restriction that nerve gaps did not exceed 1.2 cm in this study. In our cohort the time of treatment seemed to have more influence on the outcome than patient age at the time of nerve repair. The lack of motor recovery is of concern and might be caused by misdirected regrowing axons and the degeneration of motor endplates. Additional *in vitro* as well as *in vivo* trials regarding materials and cell types best suitable for conduit based nerve regeneration will hopefully enable more refined decisions regarding the optimal treatment method and selection criteria for patients suffering from large-diameter nerve lesions.
